# Limited Neutralizing Antibody Specificities Drive Neutralization Escape in Early HIV-1 Subtype C Infection

**DOI:** 10.1371/journal.ppat.1000598

**Published:** 2009-09-18

**Authors:** Penny L. Moore, Nthabeleng Ranchobe, Bronwen E. Lambson, Elin S. Gray, Eleanor Cave, Melissa-Rose Abrahams, Gama Bandawe, Koleka Mlisana, Salim S. Abdool Karim, Carolyn Williamson, Lynn Morris, the CAPRISA 002 study and the NIAID Center for HIV/AIDS Vaccine Immunology (CHAVI)

**Affiliations:** 1 AIDS Virus Research Unit, National Institute for Communicable Diseases, Johannesburg, South Africa; 2 University of the Witwatersrand, Johannesburg, South Africa; 3 Institute of Infectious Disease and Molecular Medicine, University of Cape Town, Cape Town, South Africa; 4 Centre for the AIDS Programme of Research in South Africa (CAPRISA), University of KwaZulu Natal, Durban, South Africa; University of Zurich, Switzerland

## Abstract

We previously showed that HIV-1 subtype C viruses elicit potent but highly type-specific neutralizing antibodies (nAb) within the first year of infection. In order to determine the specificity and evolution of these autologous nAbs, we examined neutralization escape in four individuals whose responses against the earliest envelope differed in magnitude and potency. Neutralization escape occurred in all participants, with later viruses showing decreased sensitivity to contemporaneous sera, although they retained sensitivity to new nAb responses. Early nAb responses were very restricted, occurring sequentially and targeting only two regions of the envelope. In V1V2, limited amino acid changes often involving indels or glycans, mediated partial or complete escape, with nAbs targeting the V1V2 region directly in 2 cases. The alpha-2 helix of C3 was also a nAb target, with neutralization escape associated with changes to positively charged residues. In one individual, relatively high titers of anti-C3 nAbs were required to drive genetic escape, taking up to 7 weeks for the resistant variant to predominate. Thereafter titers waned but were still measurable. Development of this single anti-C3 nAb specificity was associated with a 7-fold drop in HIV-1 viral load and a 4-fold rebound as the escape mutation emerged. Overall, our data suggest the development of a very limited number of neutralizing antibody specificities during the early stages of HIV-1 subtype C infection, with temporal fluctuations in specificities as escape occurs. While the mechanism of neutralization escape appears to vary between individuals, the involvement of limited regions suggests there might be common vulnerabilities in the HIV-1 subtype C transmitted envelope.

## Author Summary

Most HIV-1 infected individuals develop neutralizing antibodies against their own virus, termed an autologous neutralizing response. It is known that this response exerts pressure on the envelope of HIV, the target of such antibodies, resulting in neutralization escape. Here we have identified the targets of these antibodies and the precise genetic basis of neutralization escape in 4 individuals infected with HIV-1 subtype C. We show that V1V2 is commonly involved in escape, and that the C3 region is also a target in some cases. The latter observation confirms this region is exposed in subtype C, unlike subtype B. We show that neutralization escape is conferred by a few amino acid mutations, some of which are outside the antibody target site. Moreover, escape from these limited specificities even within a single individual occurs via a variety of different pathways involving substitutions, indels and glycan shifts. The finding in 2 individuals that an anti-C3 response developed first, followed by an anti-V1V2 response, suggests there may be specific regions of envelope particularly vulnerable to antibody neutralization. Overall, we propose a mechanistic explanation for how HIV-1 epitopes drive sequential waves of neutralization escape in early subtype C infection.

## Introduction

Neutralizing antibody (nAb) responses which target the Env of HIV-1 and block viral entry develop in most HIV-1 infected individuals, reaching detectable levels within a few months of infection when measured against the autologous Env [1],[2],[3],[4]. Much of the variation that occurs in the Env during early infection is thought to be the result of pressure exerted by autologous nAbs, which is testimony to the potency of such responses [3],[4],[5]. Neutralization escape has been documented in HIV-1 subtype B viruses [3],[4],[6],[7],[8],[9],[10],[11],[12] and in SIV [13],[14],[15] with contemporaneous viruses showing less sensitivity to autologous neutralization than earlier viruses. Even in virus controllers with relatively low levels of antigenic stimulation of B cells, continuous viral selection and escape from autologous nAbs occurs [16]. However, the dynamic nature of the autologous neutralizing response is exemplified by the fact that escape variants are sensitive to *de novo* nAb responses generated to new variants. The nature and timing of the novel responses, or whether initial autologous nAbs are maintained or decay is not clear. It seems likely that early nAbs will wane as escape occurs, when the antigen responsible for elicitation of such responses is replaced by escape variants, which would presumably no longer stimulate existing antigen-specific B cells.

Escape from autologous nAbs may occur through amino acid substitutions resulting in mutational variation at epitopes [17], insertions and deletions (indels) in the Env [18],[19], and through an “evolving glycan shield”, where a shift in the number and position of glycans prevents access of nAbs to their cognate epitopes [4],[19],[20]. The relative importance of each mechanism of escape is not clear, and in many cases, a global view of envelope mutations and indels in escaped variants has not allowed precise elucidation of the genetic basis of escape [3],[12],[17]. Furthermore, the specificities, number and kinetics of the antibodies driving escape are largely unknown.

The autologous nAb response in subtype C infection appears to differ somewhat from that in subtype B viruses and is less well-characterized. In subtype C, these antibodies develop to higher titer and are particularly type-specific with little or no cross-neutralizing activity within the first year of infection [1],[2]. The type-specificity of autologous nAbs implies that they target variable regions, and indeed we have shown that nAbs directed at the V1V2, V4 and V5 regions contributed to autologous neutralization in some HIV-1 subtype C infected individuals [1],[21].

The role of V1V2 in shielding neutralization determinants is well-recognized [19],[20],[22],[23],[24],[25],[26]. V1V2 may also act as a neutralization target in some laboratory adapted HIV isolates [27] and primary HIV isolates [1],[18],[21],[28],[29],[30],[31],[32],[33]. Furthermore, use of reciprocal V1V2 chimeras suggested that the V1V2 region was principally responsible for the strain-specific AnAbs detected in plasma from SHIV-infected monkeys [34]. In subtype C, variable regions (V1 to V4) have also been implicated in shielding neutralization determinants, and infection may be mediated by viruses with relatively short variable loops and high sensitivity to neutralization by donor sera [35]. The role of V4 and V5 in neutralization resistance is less clear, although these regions may impact on envelope conformation and glycan packing [4],[15],[36], thereby sterically limiting accessibility of neutralization determinants.

In addition to the variable regions, the C3 region, located in the outer domain of gp120 slightly upstream of the V3 loop has been implicated in neutralization escape in subtype C viruses [37]. The C3 region of subtype C viruses is under strong diversifying pressure [38] and there are distinct structural differences between subtypes B and C in the alpha 2 (α2)-helix of C3 [37] suggesting increased exposure of this region in subtype C viruses. It has therefore been proposed that nAbs directly target the α2-helix in subtype C viruses [37],[39]. We recently showed that the region is a major target of autologous neutralizing antibodies in subtype C HIV-1 infection [21].

Here we investigated neutralization escape in 4 individuals from the CAPRISA 002 Acute Infection cohort, including one virus controller, 2 individuals classified as intermediate progressors, and one rapid progressor. Neutralization profiles against the infecting virus from three of the four individuals have been described previously and were shown to differ in timing and magnitude [1],[21]. In this study, representative envelopes were derived by single genome amplification (SGA) from multiple time points during the first year of infection, and tested against autologous plasma spanning the first 2 years of infection. We examined the specificities of autologous nAbs using chimeric envelopes. Potential escape mutations were identified by examination of sequences from multiple time points, and tested by generating chimeric and/or mutant envelopes representative of polymorphisms and measuring the acquisition of neutralization sensitivity in resistant envelope clones obtained at later time-points. Our data suggest that antibodies targeting only one or two epitopes, predominantly in V1V2 and C3, drive escape in some individuals in the first year of infection in HIV-1 subtype C infection.

## Results

Previously we have shown using the CAPRISA 002 Acute Infection cohort that HIV-1 subtype C infection is characterized by the appearance in the first year of potent and highly type-specific neutralizing antibodies (nAbs) against the infecting virus [1]. In order to determine if these antibodies exert immune pressure we assessed later viruses for neutralization escape using samples from 4 individuals who were selected based on their disease profile, degree of genetic diversity and timing of the first appearance of autologous nAbs (Table 1). We hypothesized that individuals with increased genetic diversity were more likely to have multiple antibody specificities forcing genetic escape.

**Table 1:** CAPRISA 002 participants included in this study.

PTID	Clinical progression	Viral load (1 year p.i.)	CD4 count (1 year p.i.)	Genetic diversity	Timing of nAbs
		(RNA copies/ml)	(cells/µl)	(1 year p.i.)	(weeks p.i.)
CAP45	slow*	556	1,030	0.006	9
CAP88	intermediate	38,700	499	0.018	15
CAP177	intermediate	42,100	381	0.031	19
CAP210	rapid**	376,000	344	0.008	46

*: Slow progressors have a consistent viral load <2,000 RNA copies/ml.

**: Rapid progressors have CD4 count <350 cells/µl over the 1st year of infection.

Refer to Figure S1 for longitudinal viral load and CD4 count data.

### Neutralization escape in early HIV-1 subtype C infection

**Figure 1:**
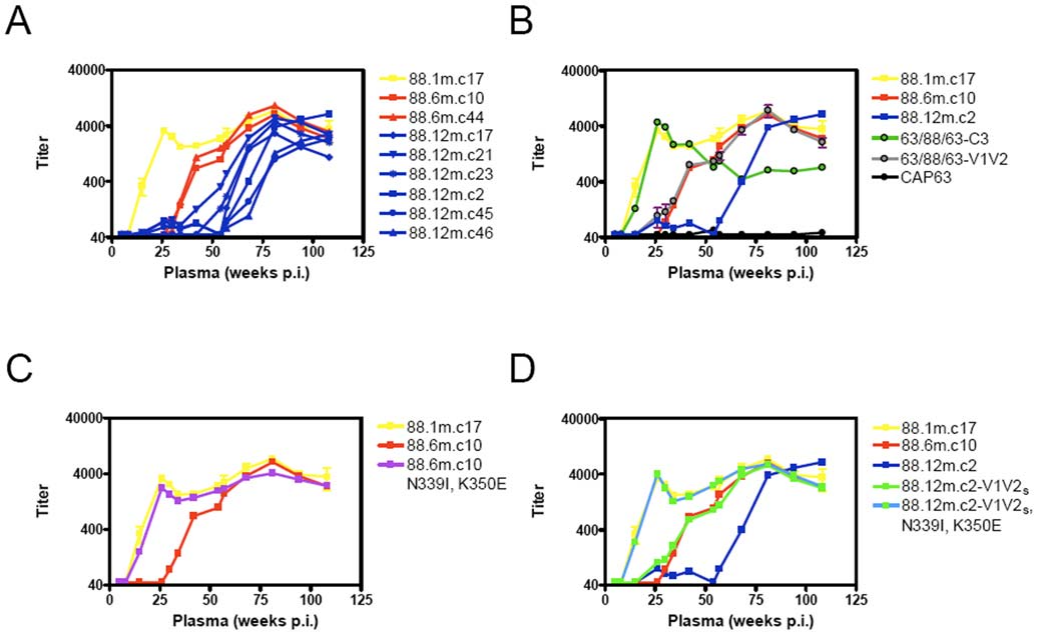
Neutralization escape in CAP88. A) Development of a biphasic autologous neutralizing response against a 1 month clone, 88.1m.c17 (yellow line) with ID_50_ titers shown on the y-axis on a logarithmic scale, and weeks post-infection of plasma on the x-axis. Neutralization escape of 6 month (red lines) and 12 month (blue lines) envelope clones was evident by rightward shifts in neutralization curves. B) Neutralization of heterologous chimeras 63-88-63-C3 (green line) and 63-88-63-V1V2 (gray line) overlaid on neutralization profiles of 6 month (red) and 12 month clones (blue) showed the evolution of distinct specificities. C) CAP88.6m.c10 N339I, K350E (purple line), showed an increase in neutralization sensitivity, shifting left to match 88.1m.c17 (yellow line). D) 88.12m.c2-V1V2_s_ (green line) and 88.12m.c2-V1V2_s_, N339I, K350E (green/blue line) showed incremental increases in neutralization sensitivity to match the 6 month clone 88.6m.c10 (red line) and 1 month clone, 88.1m.c17 (yellow line) respectively.

CAP88, an intermediate progressor, first showed a neutralizing response at 15 weeks p.i. [1]. Here we derived SGA envelope sequences from CAP88 at 1 month (enrolment), 6 months and 12 months post-infection (p.i.). Selected amplicons chosen to represent each major clade in the phylogenetic tree were cloned and tested against autologous plasma spanning the first 2 years of infection. The neutralization curve when measured using the earliest available clone, 88.1m.c17, had a biphasic shape, with one peak at approximately 26 weeks p.i. and a second peak at 81 weeks p.i. (Figure 1A). The neutralization curves for the 6 and 12 month clones were shifted incrementally to the right (Figure 1A) indicating neutralization escape. Escaped variants, however, remained sensitive to later neutralizing antibody responses as evidenced by the high titers using later plasma samples. Furthermore, the varied curves shown by the multiple 12 month clones (Figure 1A) suggested different pathways to escape.

### Temporal variations in neutralization titers correspond to waves of antibody specificities

We have previously probed the specificities of antibodies in CAP88 using heterologous chimeras, where regions of interest in CAP88 were transferred into an unrelated envelope, CAP63 [21]. Using this approach, serum from CAP88 was shown to contain at least 2 antibody specificities within the first year of infection targeting the C3 and the V1V2 regions. Here, longitudinal analysis using the heterologous chimeras over the first 2 years of infection suggested that each of the 2 peaks in the overall nAb response comprised a single specificity (Figure 1B). The neutralization curve using the heterologous chimeric envelope 63/88/63-C3 (where the C3 region from CAP88 at 1 month p.i. was transferred into the unrelated envelope, CAP63) mapped exactly to the first peak of the overall nAb response, while the neutralization curve of 63/88/63-V1V2 mapped to the second peak. These data suggested that the autologous response in CAP88 consisted of an initial response to the C3 region, peaking at 26 weeks p.i. with a titer of ∼1∶4,000, and then waning so that by 54 weeks p.i. the titer had dropped to approximately 1∶700. The second specificity targeting the V1V2 region developed from a titer of 1∶60 at 26 weeks p.i. to peak at 81 weeks p.i. with a titer exceeding 1∶5,500, thereafter it too waned to a titer of ∼1∶2,000 by 108 weeks p.i. (Figure 1B).

We were interested in the relationship between these 2 defined specificities and the neutralization escape occurring in CAP88. Comparison of the neutralization curves of representative clones from 1 month, 6 months and 12 months p.i. with the neutralization data obtained using the heterologous chimeras showed that the 1 month clone (in yellow) was sensitive to both the anti-C3 and anti-V1V2 antibodies (Figure 1B). In contrast the 6 month clone matched the second peak indicating that this clone had escaped the initial anti-C3 response but remained sensitive to the anti-V1V2 antibodies. The 12 month clones had shifted still further and had therefore escaped both the anti-C3 and anti-V1V2 responses (Figure 1B), but remained sensitive to yet another unidentified response.

### Limited amino acid changes mediate neutralization escape

**Figure 2:**
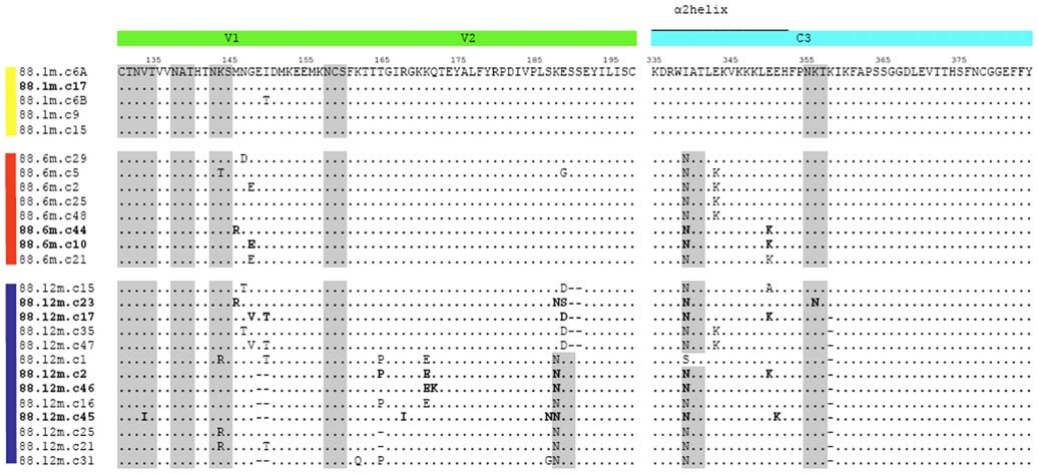
Amino acid alignment of the V1V2 (green) and C3 (blue) regions of single genome amplicons of CAP88. Amplicons were derived from 1 month p.i. (yellow bar), 6 months p.i. (red bar) and 12 month p.i. (blue bars). Amplicons highlighted in bold text were cloned for neutralization assays. Potential N-linked glycosylation sites are highlighted in gray, dashes indicate deletions.

Analysis of the C3 region of the 6 month amplicons showed that each contained 2 potential escape mutations in the α2-helix; I339N plus E343K or I339N plus E350K (Figure 2). Back mutation of the I339N and E350K changes in a representative clone, CAP88.6m.c10, to create the mutant envelope CAP88.6m.c10 N339I,K350E resulted in the resistant 6 month clone acquiring complete neutralization sensitivity, matching the 1 month clone (Figure 1C). The I339N mutation in all 6 month amplicons created a potential N-linked glycolsyation site [40],[41],[42] suggesting the possibility of glycan shielding as a mechanism of escape. However, both the E343K and E350K mutations resulted in charge switches from negatively charged glutamic acid residues to positively charged lysine residues. The charge alteration in each clone (at either position 343 or 350) may be in response to conformational changes resulting from glycosylation at the 339 residue. However mutagenesis studies in 88.6m.c10 showed that the charge change at position 350, independently of the N339 PNG site (88.6m.c10 N339I) also conferred some sensitivity (titer of 548, Table 2), though much less profoundly than the sensitivity conferred by both mutations (titer of 2,221, Table 2). This suggested that charge changes in the absence of glycans may also contribute to neutralization escape. The continuing pressure on the C3 region remained evident at 12 months p.i. where despite the waning anti-C3 levels, there was maintenance of the I339N glycosylation site, in some cases still with either E343K or E350K, plus an additional deletion in most clones at N355 (Figure 2), the significance of which is not known. Overall, by altering two amino acids in the C3 region the virus in CAP88 escaped the anti-C3 antibodies that arose within the first 6 months of infection.

**Table 2:** Role of individual escape mutations within CAP88 C3 mediating neutralization escape at 6 months p.i.

Clone ID	Amino Acid Residues		PNG	Titer at 26 weeks pi.
	339	350		
88.1m.c17	I	E	no	2,989
88.6m.c10	N	K	yes	45
88.6m.c10 N339I	I	K	no	548
88.6m.c10 N339I, K350E	I	E	no	2,221

A similar approach was used to investigate neutralization escape in the 12 month clones which were resistant to both specificities. Since the number of mutations in V1V2 precluded testing all changes by site-directed mutagenesis we used the whole V1V2 region to generate chimeras. Sequential autologous chimeras were constructed using a 12 month clone, CAP88.12m.c2, firstly introducing the V1V2 region from the sensitive early virus to create 88.12m.c2-V1V2_s_ then back-mutating the C3 region to make 88.12m.c2-V1V2_s_,N339I,K350E. Use of these chimeras in neutralization assays showed that 88.12m.c2-V1V2_s_ became sensitive to the second peak comprising anti-V1V2 antibodies, but remained resistant to anti-C3 antibodies (Figure 1D), with the neutralization curve of 88.12m.c2-V1V2_s_ matching the 6 month clone. The double chimera, 88.12m.c2-V1V2_s_,N339I,K350E shifted still further, becoming as sensitive as the earliest virus to both anti-C3 and anti-V1V2 antibodies (Figure 1D), suggesting that in this 12 month clone, escape was mediated by changes in V1V2 in addition to the maintenance of escape mutations in C3. These included a 2 amino acid deletion in V1 as well as 3 substitutions in V2. Examination of all SGA amplicons at 12 months p.i. showed the addition of a PNG in V2 in 8 of 13 amplicons, suggesting a role for glycan shielding. The remaining 5 clones all contained a 2 amino acid deletion in the same region of V2 which could perhaps re-orientate the loop resulting in ablation of the epitope (Figure 2). Therefore, the mechanism of escape from an anti-V1V2 response varied between clones present in the same individual, utilizing either deletions or glycan shielding.

### Limited nAb specificities drive sequential escape mutations

**Figure 3:**
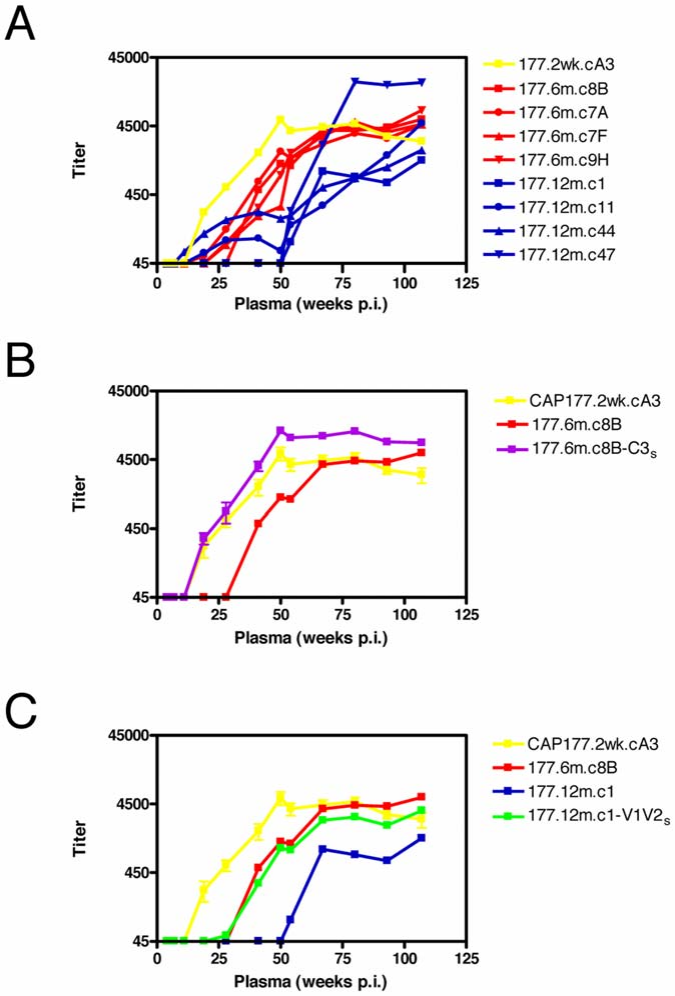
Neutralization escape in CAP177. A) Development of an autologous neutralizing response against a preseroconversion clone, 177.2wk.cA3 (yellow line) with ID_50_ titers on the y-axis, weeks post-infection of plasma on the x-axis. Neutralization escape occurred in clones from 6 months (red lines) and 12 months (blue lines) p.i. B) 177.6m.c8B-C3_s_ (purple line), a 6 month clone containing the preseroconversion C3 sequence, showed an increase in neutralization sensitivity, shifting to the left towards the preseroconversion clone, 177.2wk.cA3. C) 177.12m.c1-V1V2_s_, the 12 month clone containing the V1V2 of the preseroconversion envelope gained neutralization sensitivity, evident in a shift to the left so that the chimeric envelope matches the earlier clone, 177.6m.c8B.

The concept of evolving nAb specificities which drive sequential escape mutations was supported by data from a second individual, CAP177. Envelope SGA amplicons were derived from CAP177 at 2 weeks (preseroconversion), 6 months and 12 months p.i. Use of the transmitted envelope, inferred from the consensus sequence at preseroconversion, 177.2wk.cA3, showed the development of autologous nAbs by 19 weeks p.i., peaking at 1∶5,500 at about 50 weeks p.i. (Figure 3A). Like CAP88, clones obtained at 6 months and 12 months p.i. all exhibited neutralization escape (Figure 3A). Due to the relatively high levels of variability across the envelope we again made use of autologous chimeric viruses, where we exchanged either V1V2, C2, C3, V4 or C3-V4 (regions exhibiting changes in all 12 month clones) between the sensitive preseroconversion envelope and a representative clone from 6 months and 12 months p.i. When the C3 region from the preseroconversion envelope was transferred into a 6 month clone, 177.6m.c8B to form 177.6m.c8B-C3_s_, neutralization sensitivity increased markedly, with titers higher than those seen with 177.2wk.cA3, although the shapes of the curve were remarkably similar (Figure 3B). The increase in sensitivity of the 177.6m.c8B-C3_zs_ envelope suggested that, as with CAP88, escape initially occurred via changes in the C3 region. Four changes were noted in the C3 of 177.6m.c8B (Figure S2), one of which resulted in a shift in the position of a PNG, though the overall number of PNGs in the region remained unchanged. All four changes were charge changes from neutral or negatively charged residues to positively charged residues. This was similar to CAP88 although the fact that mutated residues differed between clones at 6 months p.i. in both CAP88 and CAP177 suggested multiple mechanisms of escape within C3 even in a single individual.

When we examined neutralization escape at 12 months p.i., transfer of the V1V2 region from the preseroconversion envelope into 177.12m.c1 to form 177.12m.c1-V1V2_s_ resulted in a shift leftwards of the neutralization curve, with the chimeric envelope becoming as sensitive to neutralization as the 6 month clone (Figure 3C). All of the 12 month clones contained at minimum a 3 amino acid insertion in the V1 loop, resulting in the addition of either one or two novel PNGs (Figure S2). It is likely that the extended loop length in addition to the novel PNGs mediated escape through shielding of neutralization epitopes. These data support the notion that waves of nAb specificities drive sequential escape mutations over the first year of infection.

### The V1V2 region may serve as a nAb target, and also directly mediate neutralization escape

**Figure 4:**
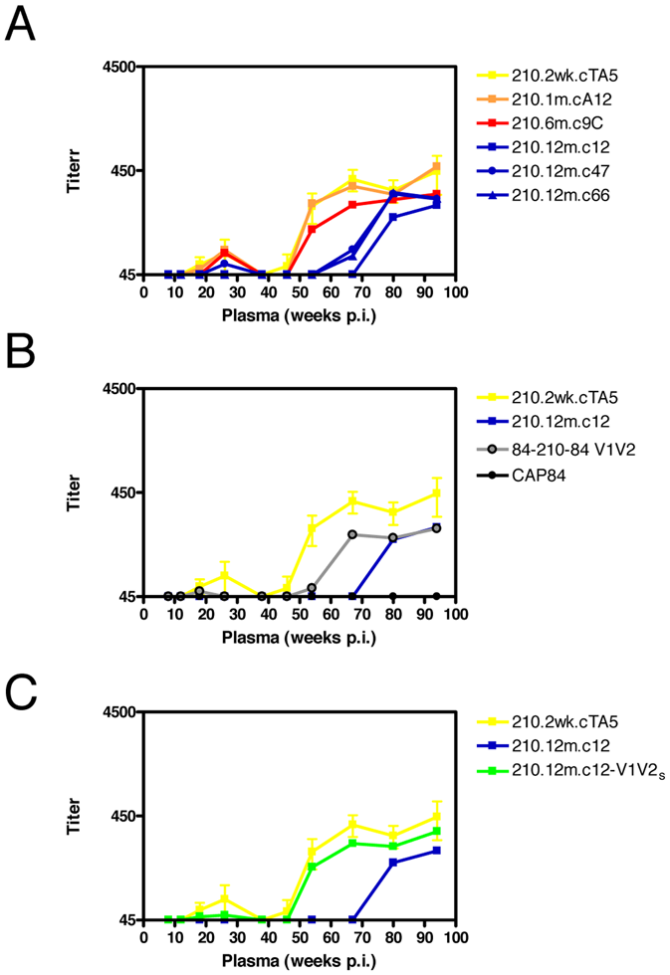
Neutralization escape in CAP210. A) Development of a delayed autologous neutralizing response against a preseroconversion clone, 210.2wk.cTA5. Clones obtained at 1 month p.i. (orange line) and 6 months p.i. (red lines) showed no neutralization escape, in contrast to clones obtained at 12 months p.i. (blue lines). B) Neutralization of the heterologous chimera 84-210-84-V1V2 (gray line) showed the development of anti-V1V2 antibodies from 54 weeks p.i. C) 210.12m.c12-V1V2_s_, a resistant 12 month clone (green line) that contained the V1V2 region from the preseroconversion envelope showed an increase in neutralization sensitivity to match the preseroconversion clone 210.2wk.cTA5 (yellow line).

A role for autologous nAbs targeting V1V2, with escape mutations occurring directly in V1V2 was also shown in CAP210. The autologous neutralizing response in CAP210, a rapid progressor, was of low magnitude and only became consistently detectable at 46 weeks p.i. when measured against the transmitted envelope (yellow) (Figure 4A). Neutralization escape in CAP210 occurred late, with the 1 month and 6 month clones showing identical sensitivity to the earlier envelope despite occasional sequence changes (Figure S3). This was unsurprising considering the absence of measurable nAbs at these time points. In contrast the 12 month clones, from a time-point very shortly after the development of measurable autologous nAbs did exhibit neutralization escape with a shift to the right in the neutralization curves. As with CAP88, we constructed a heterologous chimera for the V1V2 region (where the majority of amino acid changes were located in 12 month clones) to assess whether anti-V1V2 antibodies mediated autologous neutralization. We observed transfer of neutralization sensitivity using the heterologous 84/210/84-V1V2 chimera (where the V1V2 region from CAP210.2wk.cTA5 was transferred into the unrelated envelope CAP84) suggesting that anti-V1V2 antibodies were present in CAP210 sera from 54 weeks p.i., coinciding with the timing of detectable autologous nAbs (Figure 4B).

In order to assess whether changes in V1V2 also mediated escape in CAP210, as we had shown in CAP88 and CAP177, we created an autologous chimera, transferring the V1V2 region from the early sensitive clone into a 12 month resistant clone, 210.12m.c12. The autologous V1V2 chimeric envelope, 210.12m.c12-V1V2_s_ showed a shift left in the neutralization curve, becoming as sensitive as the early preseroconversion envelope (Figure 4C). While all CAP210 12 month SGA sequences contained an A153V mutation at the beginning of V2, the presence of this change in the sensitive 6 month clone 210.6m.cC9 and other amplicons from 6 months p.i., suggested that this mutation was not involved in neutralization escape (Figure S3). In resistant clone 210.12m.c12 there were no changes in PNG sites, although a 2 amino acid deletion at position 180 between 2 PNGs could perhaps alter the arrangement of the PNGs in this clone. Alternatively, deletion may alter the conformation or directly ablate the epitope independently of glycosylation. In contrast, clones 210.12m.c47 and 210.12m.c66 (which had superimposable neutralization curves and were slightly more sensitive than 210.12m.c12) both had the addition of a single PNG in V1 at position 132, as well as an E181K change between two PNGs. Overall, these data suggested that the initial autologous nAb response in CAP210 was comprised solely of anti-V1V2 antibodies, which drove neutralization escape via mutations within V1V2, likely via shifting glycans.

### Changes outside the antibody target may mediate escape

**Figure 5:**
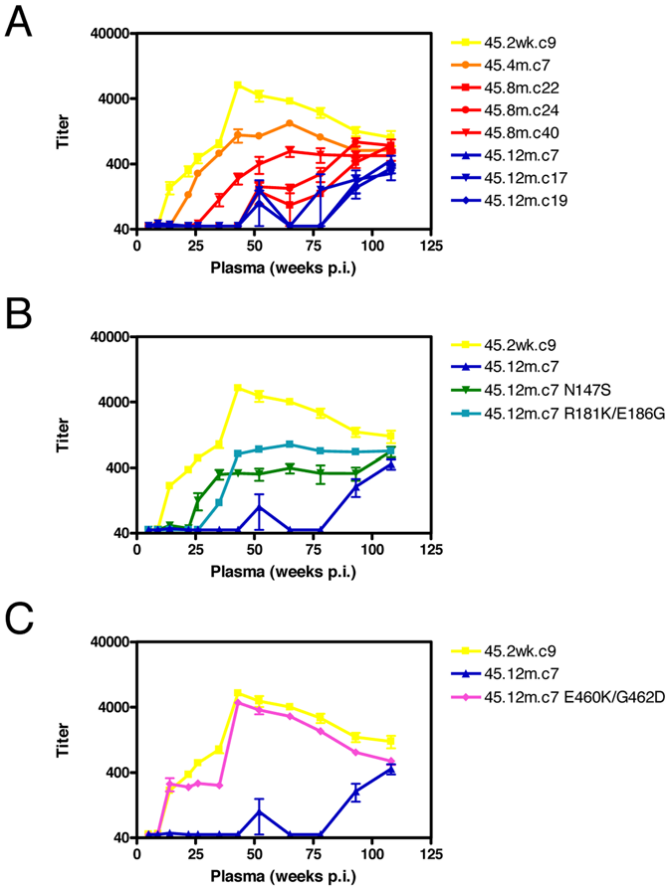
Neutralization escape in CAP45. A) Development of an autologous neutralizing response against a preseroconversion clone, 45.2wk.c9 (yellow line) with ID_50_ titers on the y-axis, weeks post-infection on the x-axis. Neutralization escape occurred in clones from 6 months (red lines) and 12 months (blue lines) p.i. B) Site-directed mutants of the escaped 12 month clone 45.12m.c7 to restore N147S in V1 (green line) and R181K/E186G in V2 (cyan line) to preseroconversion motifs resulted in slight acquisition of sensitivity compared to the resistant 12 month clone (blue line). C) Site-directed mutation of 45.12m.c7 to restore E460K/G462D in V5 (pink line) to preseroconversion motifs resulted in complete restoration of sensitivity, with the neutralization profile matching the preseroconversion clone, 45.2wk.c9 (yellow line).

In a fourth individual, CAP45, previous analyses suggested that autologous nAbs targeted C3-V4 and V1V2 [21]. CAP45 who was a slow progressor developed a robust autologous response by 9 weeks p.i. peaking at titers exceeding 1∶6,000 at 43 weeks p.i. when measured against the transmitted envelope (Figure 5A). Envelope clones obtained at 4 months, 8 months and 12 months p.i. escaped the early nAbs as shown by the increasing shift right in the neutralization curves. Inspection of SGA sequences showed potential escape mutations in V1V2, however despite data suggesting the C3-V4 region was a target, there were no changes in C3-V4 indicating that escape occurred by a more indirect mechanism. The level of genetic diversity in CAP45 was very low, and so it was possible to examine all changes using site-directed mutagenesis. We back-mutated each of the 8 changes observed in a 12 month clone, 45.12m.c7 to match the preseroconversion motifs.

Mutations in the V1V2 region contributed marginally to neutralization sensitivity in CAP45 (Figure 5B). The S147N change in V1, which resulted in the shift by one amino acid of a PNG (Figure 5B, green line and Figure S4), and the mutations observed in V2 (K181R, G186E, neither of which impacted on PNG formation) (Figure 5B, blue line) both increased neutralization sensitivity with relatively low titers of ∼1∶500 and ∼1∶900 respectively. This did not conflict with data obtained using the heterologous chimera which suggested only low level anti-V1V2 nAbs in CAP45 serum, becoming detectable at 35 weeks p.i. and not exceeding a titer of 1∶800 (data not shown). However neither of these changes in V1V2 accounted for complete restoration of neutralization sensitivity. The most significant escape mutations observed in CAP45 were located in V5 (K460E, D462G), and neither affected glycosylation. Simultaneous back-mutation of these 2 residues to match the preseroconversion motif resulted in almost complete restoration of sensitivity (Figure 5C, pink line). No other mutations observed in resistant 12 month clones impacted on neutralization sensitivity when back-mutated to preseroconversion motifs (data not shown).

Making use of heterologous chimeras described previously [21], we saw no evidence for anti-V5 antibodies in CAP45, suggesting that V5 is unlikely to be a direct target of nAbs in CAP45. It seems more likely that V5, and escape mutations therein, were responsible for exposure of epitope(s) in other regions of the envelope, perhaps in C3-V4 which is in relatively close proximity to V5 (Gnanakaran, *pers comm*), and which we proposed to be a nAb target in CAP45 [21]. Nevertheless, the existence of one significant mechanism of escape in CAP45 at 12 months p.i. suggests that, as with CAP210, a single neutralizing antibody was solely responsible for driving escape in this individual.

### How quickly does neutralization escape occur?

**Figure 6:**
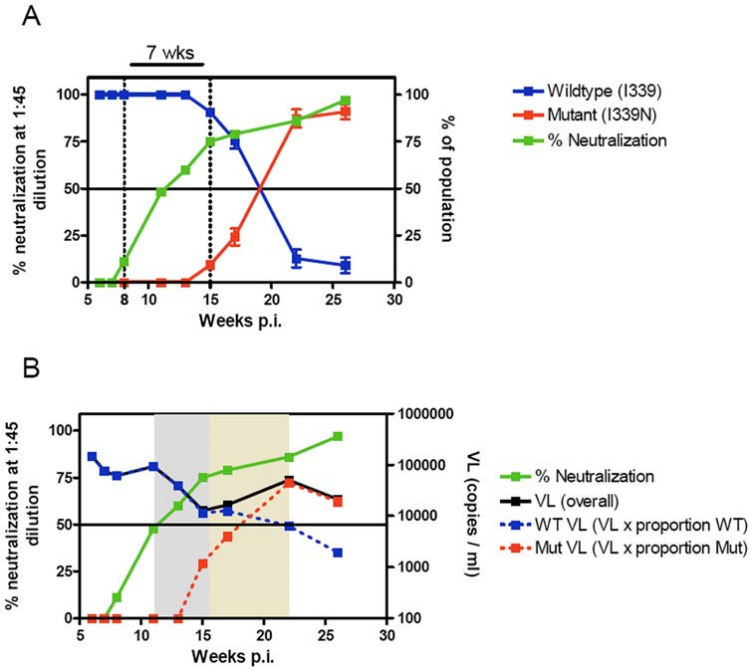
Defining the rate of neutralization escape and impact of nAbs on viral load. A) Rate of development of I339N escape mutation in CAP88. Relative proportion of wildtype I339 (blue line, right axis), escape mutant N339 (red line, right axis) and percentage neutralization at 1∶45 plasma dilution (green line, left axis) versus weeks post-infection. Relative proportions of wild-type and mutant codons were quantified using an allele-specific PCR for the 339 locus. B) Percentage neutralization at 1∶45 plasma dilution (green line, left axis), overall viral load (black line, right axis), wild type viral load (dotted blue line, right axis) and mutant viral load (dotted red line, right axis) versus weeks post-infection. Gray shading indicates a decrease in VL associated with the development of nAbs, and brown shading an increase with VL associated with neutralization escape.

Given that a single amino acid change (I339N) was associated with escape from the anti-C3 antibody in CAP88, this allowed us to measure the timing of the emergence of the escape mutation relative to the appearance of the antibody. We made use of quantitative allele-specific real-time PCR to investigate the development of the I339N change in the C3 over time. Figure 6A shows the decline in the wild-type (I339) residue from 100% to approximately 10% of the total circulating population, with a concomitant increase in the proportion of the I339N mutation by 26 weeks p.i. Interestingly, there was a 7 week gap between detectable neutralizing antibodies and the appearance of escape mutations. This suggested that relatively high levels of autologous nAbs were needed to impact on the evolution of genetic escape mutations.

Development of the anti-C3 response corresponded temporally with an 86% decrease in viral load from 93,400 RNA copies/ml at 11 weeks p.i. to 12,800 copies/ml by 15 weeks p.i. (Figure 6B). At this stage (15 weeks p.i.) 90% of circulating virus remained genotypically wild-type. The viral load rebounded to reach a level of 50,500 copies/ml at 22 week p.i., at which stage the escape mutant dominated the viral population. Extrapolation of the real-time PCR data to determine the wild-type and mutant viral load suggested that the wild-type virus continued to decline reaching levels of approximately 1,900 copies/ml at 26 weeks p.i. This suggested that the potent nAbs identified *in vitro* and through analyses of genetic escape impacted at least in the short-term on viral levels *in vivo*.

## Discussion

**Figure 7:**
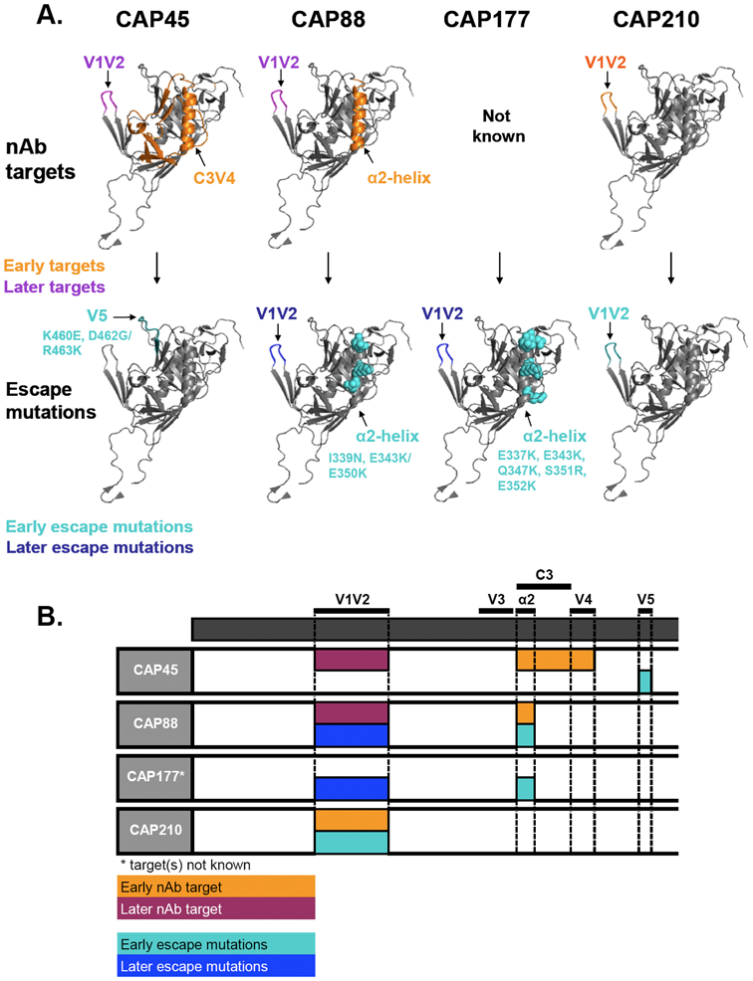
Summary of the location of the targets of autologous nAbs and resulting escape mutations. A) Location of regions targeted by nAbs on a liganded structure of gp120 [52] (with early targets shown in orange, and later targets shown in purple) and the locations of escape mutations (early mutations in cyan and later mutations in dark blue). B) a linear representation of the regions targeted by autologous nAbs and of resulting mutations showing the sequential development of specificities.

This study aimed to identify the antibody specificities mediating autologous neutralization in HIV-1 subtype C infection during the first year of infection, and investigate the mechanisms of viral escape from these antibodies. We showed that neutralization escape occurred shortly after the appearance of nAbs, and was mediated by relatively few amino acid changes, including substitutions, indels, and glycan shifts, commonly in the V1V2 and C3 regions. A limited number of specificities were responsible for driving genetic escape and these antibodies appeared sequentially and waned as escape mutations emerged. A schematic representation of the targets of autologous nAbs as well as the regions or residues involved in neutralization escape are shown in Figure 7.

The investigation of neutralization escape in conjunction with data examining the specificity of the circulating nAbs is a strength of this study. We examined neutralization escape in four individuals, with varying disease status and viral genetic diversity. In CAP45 and CAP210, where genetic diversity at 1 year p.i. was low, use of chimeric and mutant envelopes indicated the development of only a single nAb specificity resulting in neutralization escape. The low levels of viremia in CAP45 who was classified as a controller (Table 1), may have resulted in minimal antigenic stimulation of B cells, perhaps accounting for the development of only a single specificity. In contrast, in CAP210 who was a rapid progressor with a high viral load, it is somewhat surprising that the high antigenic load did not result in many more specificities and much earlier development of a nAb response. It is possible that the high viral load in CAP210 impacted on B cell dysfunction, delaying the development of nAbs (Table 1). In CAP88 and CAP177, both of whom exhibited higher genetic diversity after 1 year of infection, two distinct nAb specificities were involved in driving escape within the first year of infection. It is not clear whether the increased diversity was responsible for generating multiple antibody specificities, or whether the reverse is true. However, it was interesting to note that a very small proportion of these changes were directly involved in neutralization escape. CAP88 had an envelope CTL response targeting C2 (Clive Gray, *pers comm*) which resulted in genotypic changes in this region. Thus, nAb and CTL pressure directly accounted for 7 of the 14 mutations found in the CAP88 envelope, with the remaining 7 mutations playing no obvious role in immune escape. Thus, attributing bulk variation in envelope sequences largely to immune pressure may overstate the direct effect of early nAbs on envelope variation. Of course, it is likely that other mutations may indirectly affect neutralization sensitivity via e.g. changes in fitness or entry efficiency which could increase the overall neutralization resistance, but which should be differentiated from specific changes mediating neutralization escape from single antibody specificities

Changes within the V1V2 region were implicated in neutralization escape in all 4 individuals (Figure 7) although in CAP45 this was minor. This observation is perhaps not particularly surprising, as the role of V1V2 in shielding neutralization determinants is well-recognized [19],[20],[22],[23],[24],[25],[26]. Furthermore, we and others have proposed that the V1V2 may serve as a neutralization target in some cases [1],[18],[21]. Here we show clearly in 2 cases (CAP88 and CAP210), using heterologous chimeric viruses, evidence for nAbs which directly target the V1V2 region with neutralization escape occurring via changes within this region. Such changes were variable even within a single individual and involved multiple mechanisms. In CAP88, the unique V1V2 sequences in each of the 13 amplicons obtained at 12 months p.i. suggested that escape from the anti-V1V2 response occurred via either glycan shifts, indels or substitutions (Figure 2). Similarly, in CAP210, virtually every amplicon was unique within the V1V2 (Figure S3). Although for both CAP88 and CAP210, phenotypic testing was only performed on selected clones, the presence in plasma of these multiple variants suggests the likelihood that such sequence changes also confer escape. While changes within V1V2 also conferred neutralization escape in CAP177 we could not determine whether V1V2 was a direct antibody target in this case (Figure 7). Collectively, these observations suggest that V1V2 utilizes many pathways to escape, even when the selection pressure is induced by a single antibody specificity, almost certainly reflecting the extreme plasticity of the V1V2 region.

We previously showed that the C3-V4 region is a major target of autologous nAbs in subtype C [21]. In general, the α2-helices of subtype C viruses have more defined polar and non-polar faces than those in subtype B which are more hydrophobic [37]. This amphipathicity, characteristic of surface helices, suggests that the α2-helix in subtype C may be more exposed. Indeed, it has been proposed that nAbs directly target the α2-helix in subtype C viruses, a possibility supported by our recent data [21]. Furthermore, the C3 region of the HIV-1 subtype C envelope, including mutational patterns within the α2-helix, has been implicated in neutralization resistance [39]. Here we show that in CAP88 and CAP177, neutralization escape from anti-C3 nAbs was mediated by changes in the α2-helix of the C3 region (Figure 7). In CAP88 we were able to confirm that the C3 region was a direct target of nAbs. However, in both cases, neutralization escape was associated with charge changes within the α2-helix, all of which were located in the solvent-exposed portions of the helix maintaining the amphipathic structure. Neutralization escape in these 2 individuals was associated with an overall increase in positively charged residues within the α2-helix. While switching between oppositely charged residues within the α2-helix has been proposed to be mediate immune escape in subtype C viruses [37],[39], the precise mechanism whereby charge changes abrogate neutralization is unclear. Charge changes may simply disrupt the electrostatic interactions between nAbs and their epitopes if the C3 is a direct target, as we have shown in CAP88. Alternatively, since there are strong interactions between the N-terminus of the α2-helix and the C-terminus of the V4 region [37], with the conservation of a charge anti-correlation between the two regions, charge changes within the α2-helix may affect the conformation of the V4 loop with respect to the α2-helix, affecting the exposure of nAb targets in the C3 region or elsewhere.

The sequential development of nAb specificities, sometimes requiring months of infection was intriguing considering that the targets of these antibodies may have been present in the infecting virus. This is most clearly exhibited in CAP88 where an initial anti-C3 response developed, waned and was subsequently replaced by an anti-V1V2 response. The V1V2 epitope that was the target of this secondary response was present in the earliest virus, cloned at 1 month p.i. Despite the ongoing presentation of this epitope, an anti-V1V2 response was only detectable at 26 weeks p.i., 11 weeks after the detection of the initial anti-C3 response, suggesting the possibility of an immunological hierarchy. Delayed development of selected responses would be in line with the hierarchical binding antibody responses which develop in the very early stages of infection [43], prior to the development of nAbs. It is also possible that other changes which develop across the entire envelope during the first 6 months of infection affected the conformation and exposure of the secondary nAb target, V1V2, facilitating presentation of this region to the immune system only at a later time-point.

Of interest was the observation that after escape occurred, although specific responses waned, they continued to be maintained at somewhat lower levels. This was difficult to discern when looking at overall neutralization levels as novel responses replaced waning responses. However, the use of heterologous viruses in CAP88 clearly showed that the anti-C3 response was maintained, albeit at lower levels (declining from a peak of >1∶3,000 to stabilize at approximately 1∶700 during the second year of infection). How this response was maintained is not clear as by 6 months p.i., 8/8 envelope clones contained escape mutations which presumably no longer stimulated the B cells responsible for producing anti-C3 antibodies. It is possible that even after escape has occurred, low levels of sensitive variants remain in the lymph nodes and stimulate maintenance of low levels of antibodies. The persistence of such sensitive variants in the face of potent nAbs as reported by Mahalanbis *et al* [16], may result from continuous reversion of less fit escape variants, or persistent release of pre-escape variants from cell reservoirs. Alternatively, long-lived memory or plasma B cells may be responsible for the maintenance of specific responses in the absence of antigenic stimulation.

The identification of a single amino acid change associated with initial neutralization escape in CAP88 afforded us the opportunity to investigate the kinetics of the development of neutralization escape with respect to the timing of the autologous neutralization response. The relationship between percentage neutralization at a 1∶45 plasma dilution and development of the initial escape mutation, showed a lag of 7 weeks in the development of genetic escape. This suggested a threshold requirement, whereby relatively high titers of nAbs were needed before sufficient pressure was exerted on the overall population, forcing escape to occur. Maturation of the antibody response, in terms of affinity and avidity may also play a role in the duration of time required for escape to occur. The decrease in the viral load which occurred as the autologous neutralizing antibody response developed was intriguing, suggesting the possibility that autologous nAbs may in the short term impact on viral load, with this effect abrogated by the development of neutralization escape mutations.

The observation of the sequential development of anti-C3 antibodies followed by anti-V1V2 antibodies suggests that both of these regions are exposed and immunogenic on the HIV-1 subtype C envelope, possibly due to unique structural features of this viral subtype. Further studies are needed to determine if this is a common pattern, and whether emergence of these autologous antibodies is associated with decreases in HIV-1 viral load as seen in one individual who developed an anti-C3 specific nAb. However, as shown here, escape variants emerged as a result of a few genetic changes, not unlike the scenario with anti-retroviral monotherapy. The ease with which escape occurred, and the multiple pathways used to escape autologous responses further supports the notion that these responses, while driving considerable variation in the envelope region, have no long-term role in containing viral replication. Nonetheless, neutralization escape is reflective of active and ongoing replication in the face of an evolving and initially very low titer response, considerably different to a possible future vaccine scenario with pre-existing antibodies. Overall, these data provide insight into how a focused antibody response targeting limited regions of envelope in early subtype C infection drives sequential waves of neutralization escape.

## Materials and Methods

### Ethics statement

The CAPRISA Acute Infection study was reviewed and approved by the research ethics committees of the University of KwaZulu-Natal (E013/04), the University of Cape Town (025/2004), and the University of the Witwatersrand (MM040202). All participants provided written informed consent for study participation.

### Participants

Participants were from the CAPRISA 002 Acute Infection study, a cohort of 245 high risk HIV negative women which was established in 2004 in Durban, South Africa for follow-up and subsequent identification of HIV seroconversion [44]. The 4 individuals studied here included one controller (CAP45), one rapid progressor (CAP210) and 2 individuals classified as intermediate progressors (CAP88 and CAP177). Clinical profiles indicating viral loads and CD4 counts of each are shown in Figure S1. CAP45, CAP177 and CAP210 were all infected by single transmitted variants [45], with the transmitted envelope sequence inferred from the consensus sequence at the earliest available timepoint.

### Cell lines

The JC53bl-13 cell line, engineered by J. Kappes and X. Wu, was obtained from the NIH AIDS Research and Reference Reagent Program. 293T cells were obtained from Dr George Shaw (University of Alabama, Birmingham, AL). Both cell lines were cultured in D-MEM (Gibco BRL Life Technologies) containing 10% heat-inactivated fetal bovine serum (FBS) and 50 ug/ml gentamicin (Sigma). Cell monolayers were disrupted at confluency by treatment with 0.25% trypsin in 1 mM EDTA.

### Single genome amplification and sequencing

HIV-1 RNA was purified from plasma using the Qiagen Viral RNA kit, and reverse transcribed to cDNA using Superscript III Reverse Transcriptase (Invitrogen, CA). The *env* genes were amplified from single cDNA copies [46] and amplicons were directly sequenced using the ABI PRISM Big Dye Terminator Cycle Sequencing Ready Reaction kit (Applied Biosystems, Foster City, CA) and resolved on an ABI 3100 automated genetic analyzer. The full-length *env* sequences were assembled and edited using Sequencher v.4.0 software (Genecodes, Ann Arbor, MI). The number of potential N-linked glycosylation sites (PNGs) was determined using N-glycosite (http:/www.hiv.lanl.gov/content/hiv-db/GLYCOSITE/glycosite.html). Multiple sequence alignments were performed using Clustal X (ver. 1.83) and edited with BioEdit (ver. 5.0.9). Pairwise DNA distances were computed using Mega 4 [47].

### Cloning gp160 and production of pseudoviruses

Selected amplicons were cloned into the expression vector pCDNA 3.1 (directional) (Invitrogen) by re-amplification of SGA first-round products using Phusion enzyme (Finn Enzymes) with the EnvM primer [48] and directional primer, EnvAdir [21]. Env-pseudotyped viruses were obtained by co-transfecting the Env plasmid with pSG3ΔEnv [4] using Fugene transfection reagent (Roche) as previously described [1].

### Generation of chimeras and mutant envelopes

Chimeric Env were created using an overlapping PCR strategy with the inserts and flanking regions amplified in separate reactions. After linkage, the 3 Kb chimeric PCR fragments, generated using EnvAdir and EnvM primers [48], were cloned into the pCDNA 3.1 (directional) (Invitrogen) and screened for function as previously described [49]. Chimerism was confirmed by sequence analysis. Site-directed mutagenesis was performed using the Stratagene QuickChange II kit (Stratagene)

### Neutralization assays

Neutralization was measured as described previously [1] by a reduction in luciferase gene expression after single round infection of JC53bl-13 cells with Env-pseudotyped viruses [50]. Titers were calculated as the reciprocal plasma dilution (ID_50_) causing 50% reduction of relative light units (RLU).

### Quantitative real-time PCR

Real-time PCR was performed on RNA extracted from sequential plasma samples of CAP88 using the QIAamp Viral RNA Mini Kit (Qiagen). HIV-1 RNA was reverse transcribed to cDNA using the Superscript III Reverse Transcriptase System (Invitrogen) using the primer OFM19 as described [51]. cDNA was used in real-time PCR, making use of the following primers designed to detect the 339I and 339N residues: 88AS-PCR-T-for (CAT TAC TAA AGA CAG ATG Gtt) for detection of the 339I, 88AS-PCR-A-for (CAT TAC TAA AGA CAG ATG Gta) for the detection of 339N. Control primers were 88AS-PCR-control (GAG ATA TAA GAC AAG CAC ATT G) and 88AS-PCR-A/T-rev (CTA TGT GTT GTA ACT TCT AGG). The reaction was performed using ABI PowerSYBR Green PCR master mix, in the ABI 7500 Real Time PCR System. Primer concentrations were 300 nM, final volume 25 ul. Cycling was performed as follows: 95°C for 10 minutes followed by 45 cycles of 95°C for 15 seconds, 60°C for 15 seconds, and 72° for 1 minute, for a total of 45 cycles. Relative quantification of mutation frequency was determined by calculating the number of copies of wild type and mutant populations relative to an internal control.

## Supporting Information

Figure S1 Clinical profiles of CAP45, CAP88, CAP177 and CAP210. Viral load (copies/ml) in red and CD4 count (cells/µl) in blue. Clinical status for each individual is indicated in parentheses. Arrows indicate time points at which SGA amplicons were derived.
(0.19 MB TIF)

Figure S2 Clinical profiles of CAP45, CAP88, CAP177 and CAP210. Viral load (copies/ml) in red and CD4 count (cells/µl) in blue. Clinical status for each individual is indicated in parentheses. Arrows indicate time points at which SGA amplicons were derived.
(0.47 MB TIF)

Figure S3 Amino acid alignment of the V1V2 regions of single genome amplicons of CAP210. Amplicons were derived from 1 month p.i. (yellow bar), 1 month p.i. (orange bar), 6 months p.i. (red bar) and 12 month p.i. (blue bar). Amplicons highlighted in bold text were cloned for neutralization assays. Potential N-linked glycosylation sites are highlighted in gray, dashes indicate deletions.
(0.24 MB TIF)

Figure S4 Amino acid alignment of the mutation observed in V1 (green), V2 (cyan) and V5 (pink) regions of single genome amplicons of CAP45. Amplicons were derived from 2 weeks p.i. (yellow bar), 1 month (orange), 6 months (red bar) and 12 months p.i. (blue bar). Amplicons highlighted in bold text were cloned for neutralization assays. Potential N-linked glycosylation sites are highlighted in gray, dashes indicate deletions.
(0.23 MB TIF)
