# Supplementary figures and images for: Limited Neutralizing Antibody Specificities Drive Neutralization Escape in Early HIV-1 Subtype C Infection
Source: PLoS Pathog. 2009 Sep 18;5(9):e1000598. doi: 10.1371/journal.ppat.1000598 (PMC2742164; doi:10.1371/journal.ppat.1000598)

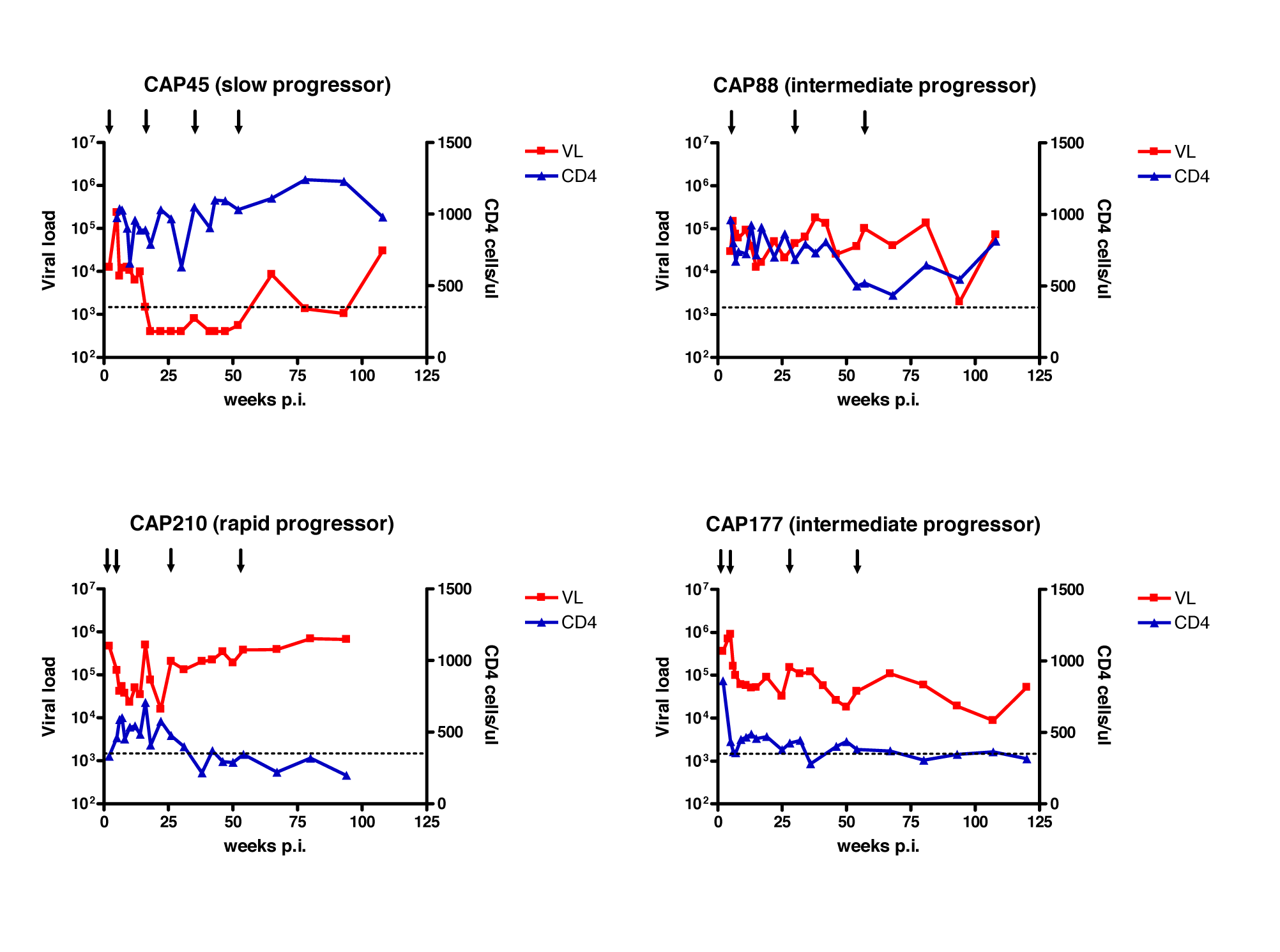

Supplement: Figure S1 — Clinical profiles of CAP45, CAP88, CAP177 and CAP210. Viral load (copies/ml) in red and CD4 count (cells/µl) in blue. Clinical status for each individual is indicated in parentheses. Arrows indicate time points at which SGA amplicons were derived. (0.19 MB TIF) [file ppat.1000598.s001.tif]

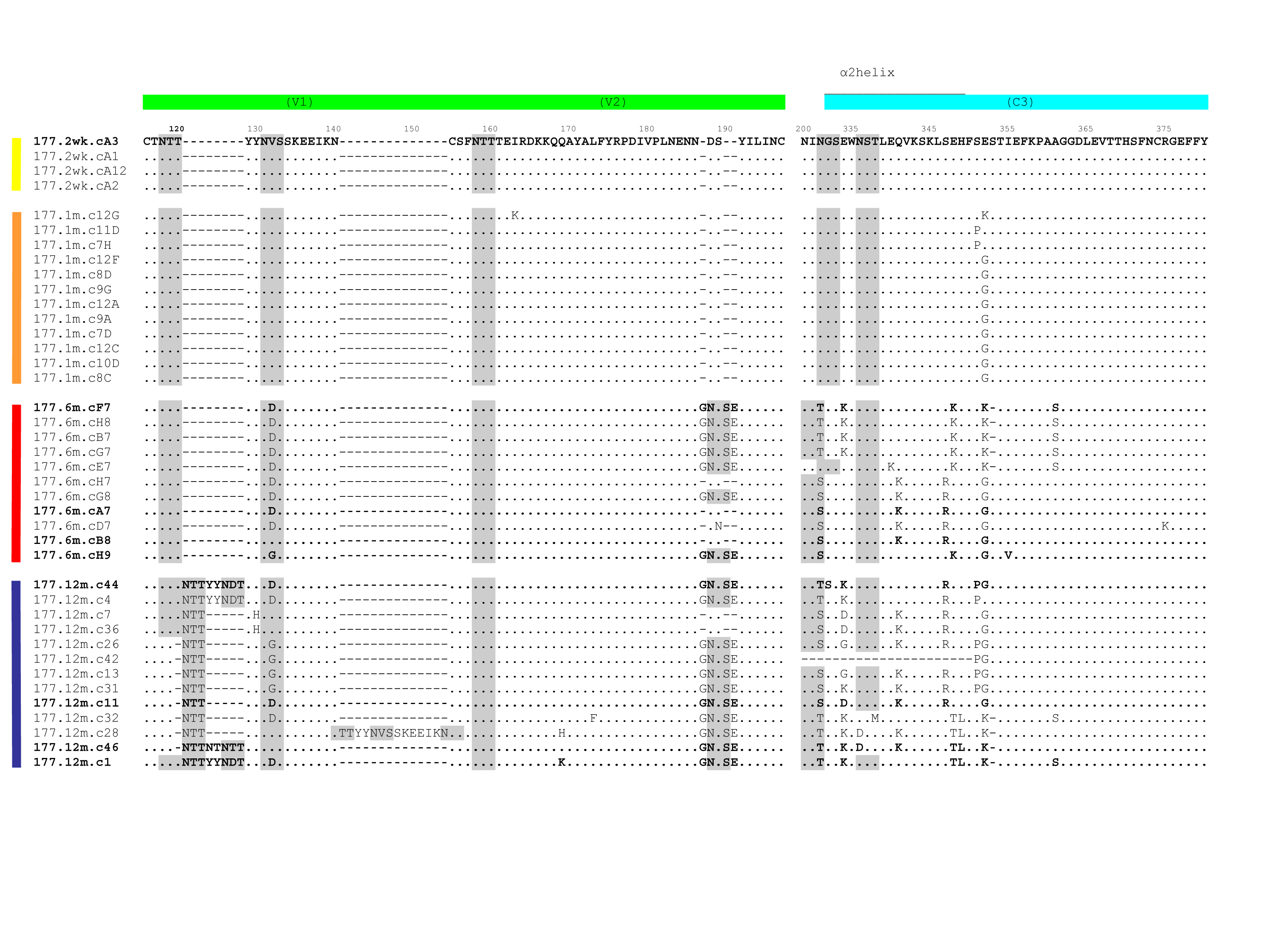

Supplement: Figure S2 — Clinical profiles of CAP45, CAP88, CAP177 and CAP210. Viral load (copies/ml) in red and CD4 count (cells/µl) in blue. Clinical status for each individual is indicated in parentheses. Arrows indicate time points at which SGA amplicons were derived. (0.47 MB TIF) [file ppat.1000598.s002.tif]

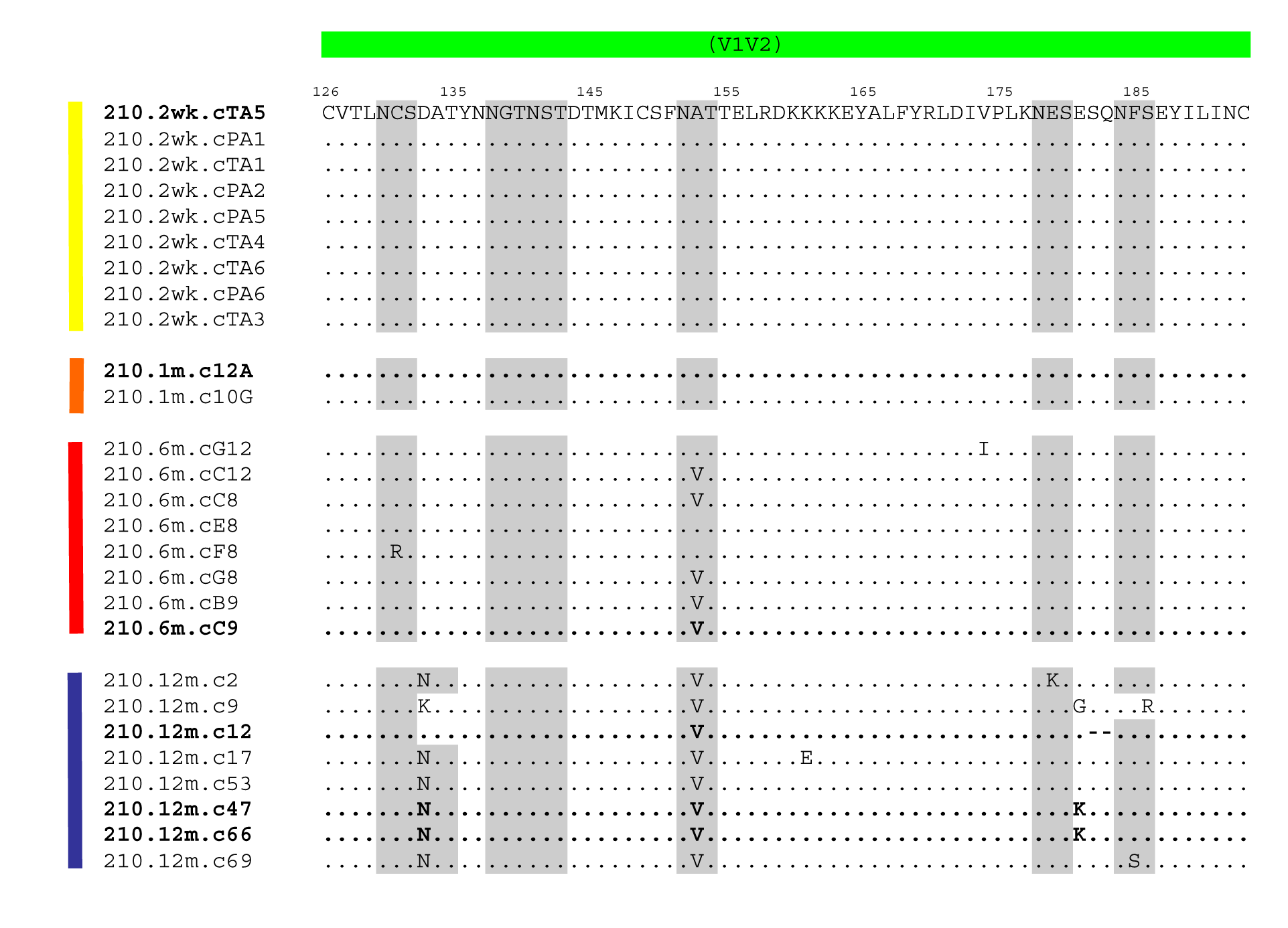

Supplement: Figure S3 — Amino acid alignment of the V1V2 regions of single genome amplicons of CAP210. Amplicons were derived from 1 month p.i. (yellow bar), 1 month p.i. (orange bar), 6 months p.i. (red bar) and 12 month p.i. (blue bar). Amplicons highlighted in bold text were cloned for neutralization assays. Potential N-linked glycosylation sites are highlighted in gray, dashes indicate deletions. (0.24 MB TIF) [file ppat.1000598.s003.tif]

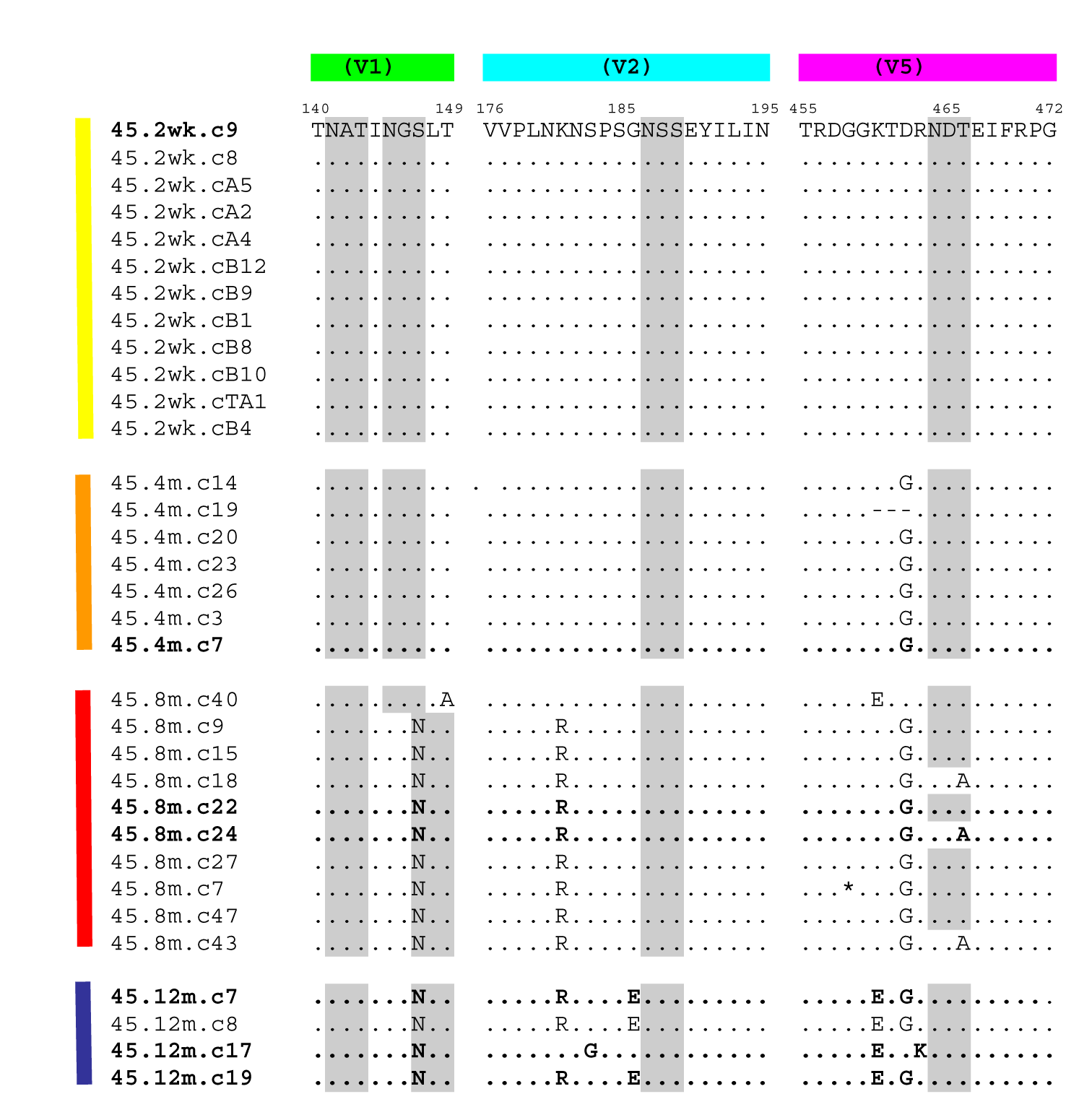

Supplement: Figure S4 — Amino acid alignment of the mutation observed in V1 (green), V2 (cyan) and V5 (pink) regions of single genome amplicons of CAP45. Amplicons were derived from 2 weeks p.i. (yellow bar), 1 month (orange), 6 months (red bar) and 12 months p.i. (blue bar). Amplicons highlighted in bold text were cloned for neutralization assays. Potential N-linked glycosylation sites are highlighted in gray, dashes indicate deletions. (0.23 MB TIF) [file ppat.1000598.s004.tif]
